# Device-associated healthcare-associated infections in an intensive care unit in Türkiye: trends and additional costs - a retrospective propensity matched cohort analysis

**DOI:** 10.1186/s12913-026-14833-3

**Published:** 2026-06-03

**Authors:** Osman Faruk Bayramlar, Selma Karabey

**Affiliations:** Department of Public Health, Istanbul Medical Faculty, Istanbul University, Istanbul, Türkiye

**Keywords:** Device-associated healthcare-associated infection, Healthcare-associated infection, Cost analysis, Intensive care unit, Propensity score matching

## Abstract

**Background:**

The additional costs of healthcare-associated infections (HAIs) impose a significant burden on healthcare systems, limiting resources for other critical health priorities. This study evaluated device-associated healthcare-associated infections (DA-HAIs) in an adult intensive care unit (ICU) and examined their associated costs.

**Materials and methods:**

A 1:1 case-control matching was performed using a propensity score matching (PSM) model to assess hospital-acquired infection costs. Data from 700 hospitalizations between 2016 and 2018 at the Anesthesiology and Reanimation ICU of Istanbul University Istanbul Medical Faculty were analyzed. Confounding factors were identified using multivariable linear regression, and PSM was applied to account for case-control differences. DA-HAI incidence and invasive device utilization over the past 15 years since the start of routine surveillance were also examined.

**Results:**

HAIs occurred in 99 (14.1%) hospitalizations, 18 involving multiple infections. Using PSM, 81 HAI cases were optimally matched. The additional costs of ICU stays due to DA-HAIs were $6,781 for ventilator-associated pneumonia (VAP) (*n* = 26, *p* ≤ 0.001), $2,037 for central line-associated bloodstream infections (CLABSI) (*n* = 25, *p* > 0.05), $5,320 for catheter-associated urinary tract infections (CAUTI) (*n* = 12, *p* = 0.001), and $4,213 for all HAIs (*n* = 81, *p* ≤ 0.001).

**Conclusion:**

DA-HAIs substantially increased ICU stays and generated substantial additional costs. DA-HAI incidence densities were at the highest levels according to NHSN criteria.

**Supplementary information:**

The online version contains supplementary material available at 10.1186/s12913-026-14833-3.

## Introduction

Healthcare-associated infections (HAIs) increase the length of stay (LoS) and hospitalization costs, elevate morbidity and mortality, and reduce the quality of life. These factors are key indicators of healthcare quality. In developed countries, HAI rates are considered one of the best markers of healthcare quality [1].

For an effective infection control program, accurate measurements must be made. One important metric is the cost associated with these infections. Investigating the direct economic impact of HAIs not only helps assess the value of prevention and control efforts but also highlights the financial burden these infections impose on healthcare systems.

Early economic evaluations of HAIs highlighted their substantial financial burden and laid the methodological foundations for matched cost analyses [2–5].

Many authors have highlighted the economic burden of HAIs, yet considerable heterogeneity exists in cost estimates and methodological approaches across studies [6, 7]. A major source of this variability is inadequate control of confounding and time-dependent bias in observational analyses of healthcare-associated infections. Advanced statistical approaches such as propensity score matching (PSM) have been recommended to improve causal inference in non-randomized settings [8]. However, their application in device-associated HAI cost analyses remains limited. In this context, our study applies a PSM framework to better address confounding in estimating the economic impact of DA-HAIs.

This study aims to calculate HAIs and device-associated healthcare-associated infections (DA-HAIs) rates among patients admitted to a university hospital intensive care unit (ICU) in Türkiye, as well as the additional costs generated by these infections.

## Material and method

### Study design and population

This observational, analytical, retrospective cohort study was conducted between 2016 and 2018 in the ICU of the Istanbul University Istanbul Faculty of Medicine, Department of Anesthesiology and Reanimation [9]. This university hospital, a tertiary healthcare institution with a capacity of 1,500 beds, is strategically located in one of the most central areas of Türkiye’s most populous metropolis. The ICU itself is a tertiary adult unit with 15 beds, including one isolation room, where targeted, active, and prospective surveillance has been performed since 2005.

### Inclusion criteria

1. Completion of the patient surveillance form, consisting of 25 questions developed by the hospital, which encompasses sociodemographic data, risk factors, comorbidities, and other relevant information for patients in the ICU (Supplemental Figure 1). This form is filled out regularly on a daily basis by infection control nurses for all patients in the ICUs.
2. Patients must be 18 years of age or older.
3. Patient insurance must be covered by the Social Security Institution.
4. Patients must have at least 48 hours of ICU LoS.
5. Patients must have been discharged or deceased between 2016 and 2018.

### Sample

A total of 764 hospitalization files were analyzed over three years, with 700 meeting the inclusion criteria. All inpatients who developed HAIs, confirmed by the infection control team, were selected as cases. Patients without HAIs were matched one-to-one using the PSM technique to eliminate bias in the comparative analysis. The study focused on DA-HAIs, specifically examining three primary groups: ventilator-associated pneumonia (VAP), central line-associated bloodstream infection (CLABSI), and catheter-associated urinary tract infection (CAUTI).

**Fig. 1:**
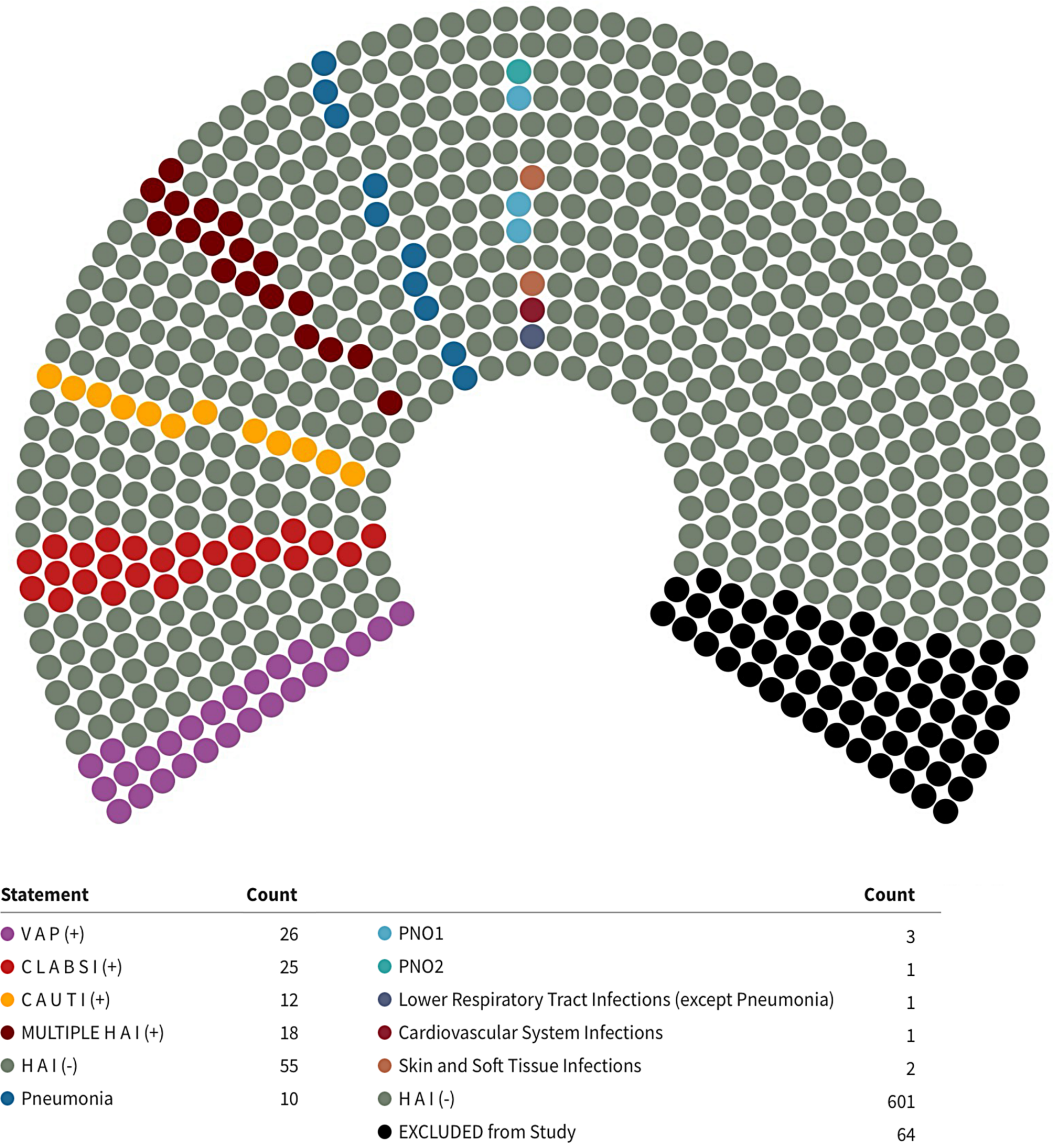
Distribution of 700 patients with hospitalization data belonging to the ICU according to HAI status surveillance. **PNO1:** Microbiologically confirmed pneumonia. **PNO2**: Clinically defined pneumonia without microbiological confirmation

### Control groups

Patients without HAIs were included in the control pool (shown as HAI (-) in Fig. 1, *n* = 601), while specific control pools were established for DA-HAIs: Patients on mechanical ventilation (MV) were assigned to the VAP control pool (*n* = 478), those with central venous catheters (CVCs) to the CLABSI control pool (*n* = 503), and those with urinary catheters to the CAUTI control pool (*n* = 579). From these four control pools, the most quantitatively and qualitatively suitable patients were selected to match the case groups one-to-one using PSM (n^HAI^: 81:81, n^VAP^: 26:26, n^CLABSI^: 25:25, and n^CAUTI^: 12:12)

### Controlling the confounding factors

Due to the observational nature of our study, randomization was not feasible and confounding could not be eliminated by design. Therefore, we identified potential confounding variables a priori based on the literature and clinical plausibility.

Isolation status and length of stay (LoS) have consistently been identified as major cost drivers in HAI studies [10, 11]. In addition, failure to account for pre-infection exposure time may introduce time-dependent bias in observational analyses of healthcare-associated infections [12]. Accordingly, pre-infection exposure time was carefully considered in our analytical framework.

For device-associated HAIs, device utilization duration was treated as a clinically meaningful exposure variable. In accordance with institutional infection control protocols, device use was discontinued upon diagnosis of infection whenever clinically feasible. Device duration was therefore calculated up to infection onset to reflect pre-infection exposure and to minimize time-dependent bias.

Importantly, prolonged device utilization represents part of the causal pathway leading to device-associated infections rather than a purely baseline confounder. Therefore, while device duration was incorporated into the analytical framework, complete balancing of exposure duration between groups was not enforced, in order to avoid over-adjustment and preserve etiological interpretability.

To identify potential confounders, we first conducted correlation analyses among candidate variables. Multivariable linear regression was performed to explore determinants of cost, and multivariable logistic regression was used to identify predictors of HAI development. Variables demonstrating strong collinearity (e.g., tracheostomy and peripheral artery catheter use) were excluded from the final propensity score model.

**Fig. 2:**
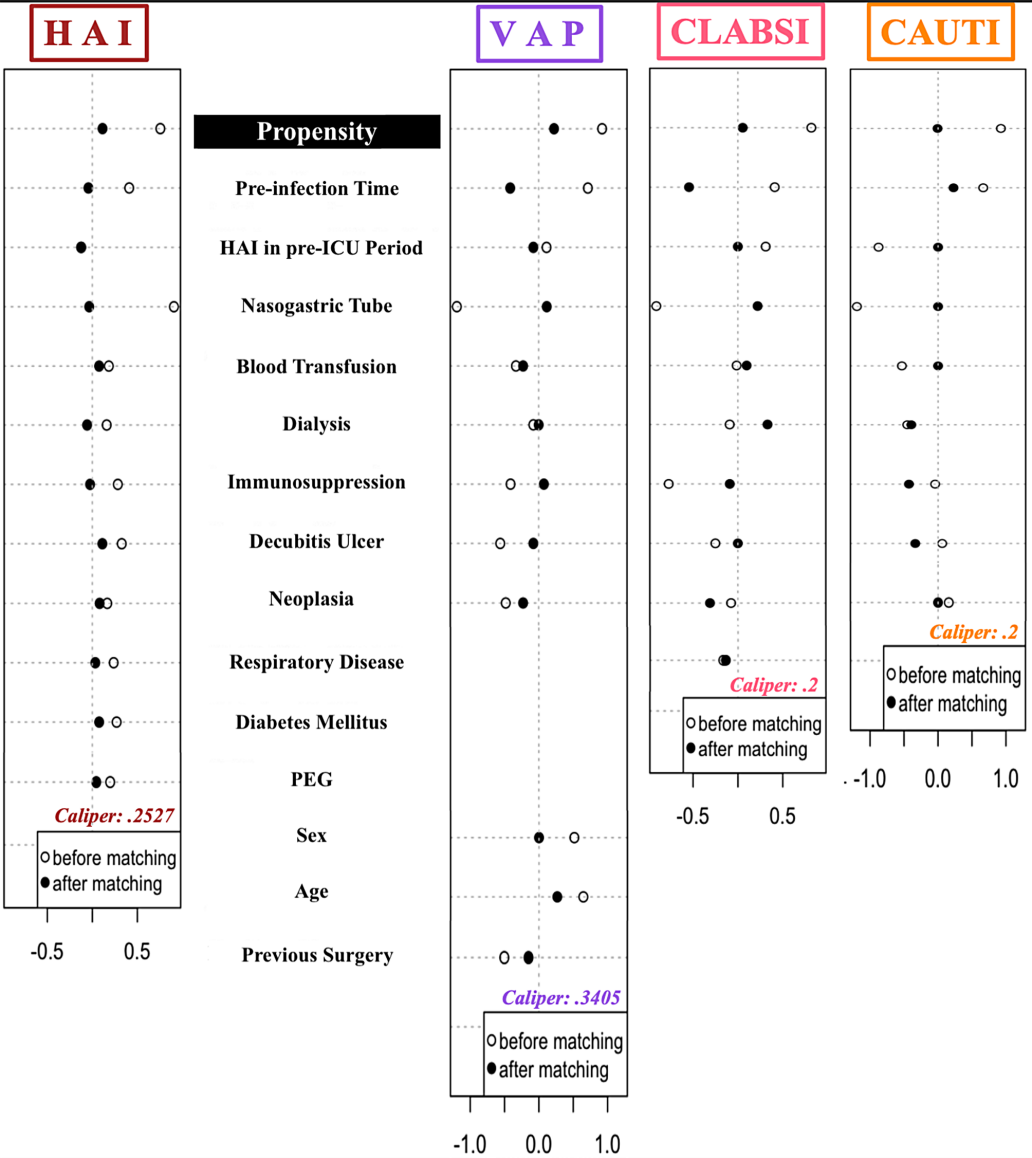
Standardized mean differences (SMDs) of baseline covariates before and after propensity score matching for HAI, VAP, CLABSI, and CAUTI analyses

Propensity scores were estimated using 14 clinically relevant covariates (Fig. 2). Nearest-neighbor matching was performed with an optimized caliper width to achieve adequate covariate balance while preserving sample size. Covariate balance was assessed using standardized mean differences (SMD), with values closer to zero indicating improved balance after matching. No DA-HAI cases were excluded during propensity score matching; matching primarily restricted the control group to patients exposed to the corresponding device.

### DA-HAI definitions

DA-HAIs were identified using the surveillance definitions of the Centers for Disease CDC NHSN Patient Safety Component Manual applicable during the study period. VAP was defined as pneumonia occurring in patients receiving mechanical ventilation for ≥48 hours and meeting the radiographic, clinical, and microbiological criteria specified in the NHSN surveillance definitions. CLABSI and CAUTI were defined according to the corresponding NHSN criteria [13]. All infections were assessed using standardized NHSN diagnostic algorithms and recorded through routine infection surveillance procedures conducted by the hospital infection control team.

### Cost data

To assess hospitalization details and costs, data were collected through the HIMS. This included patient admission and discharge dates, ICU stay specifics, social security information, ward transfers before and after the ICU, total hospitalization costs, and Social Security Institution bills for the entire ICU stay.

The total direct ICU costs included bed-day charges, medical and nursing services, laboratory and imaging procedures, pharmacological treatments, medical consumables, and isolation-related surcharges as recorded in the hospital billing system. Post-ICU ward costs were not separately analyzed.

All cost calculations were originally performed in Turkish Lira (TRY) according to the official reimbursement tariffs of the Social Security Institution applicable during the study period (2016–2018). For international comparability, cost values were converted to United States Dollars (USD) using the annual mean exchange rate for the corresponding year as reported by the Central Bank of the Republic of Türkiye [14]. All reported values represent nominal costs and were not adjusted for inflation or purchasing power parity.

The study focused on the additional burden imposed by the total direct costs of HAIs, excluding fixed costs such as salaries, utilities, and cleaning services, which account for up to 85% of hospital expenses [15]. This exclusion allowed for a clearer analysis of cost variances directly attributable to HAIs.

### Course of the ICU with percentile cut-offs

Surveillance data were available from 2005 onward, representing the earliest accessible dataset in our institution. However, complete data were unavailable for the years 2009–2011 and part of 2013 due to institutional data system transitions and archival limitations. These predefined periods were therefore excluded from longitudinal trend analyses. No additional historical data were missing beyond these specified intervals. Cutoff percentiles from the following sources were compared with the study data:

1. **National Healthcare Safety Network (NHSN):** Reports from NHSN provide information and percentile figures regarding DA-HAI utilization rates for international comparisons. However, NHSN has not published reports beyond 2013 [16]. For years beyond 2013, the last publicly available NHSN percentile thresholds (2013 report) were applied as fixed benchmark values in order to ensure longitudinal comparability across the study period.
2. **The International Nosocomial Infection Control Consortium (INICC):** INICC’s HAI rates report for 19 cities in Türkiye, covering 2003 to 2012, provides DA-HAI percentile cut-off points for a decade, based on “medical/surgical” sections [17].
3. **National Nosocomial Infection Surveillance Network** (**NNIS) in Türkiye:** National percentile cut-offs for Türkiye were derived from this source [18].

Percentile scores are categorized into six ranges: 0–10^th^, 10^th^-25^th^, 25^th^-50^th^, 50^th^-75^th^, 75^th^-90^th^, and 90^th^-100^th^. For presentation, the percentile point at which the data last passed is noted (e.g., 90^th^-100^th^ is indicated as the 100^th^ percentile).

### Formulas used

1. Incidence Rate (IR)
2. Incidence Density (ID)
3. Device Utilization Ratio [19, 20]

### Statistical analysis

Qualitative variables were compared using the Pearson Chi-square and Fisher’s exact tests. The Kruskal-Wallis test was employed for analyzing continuous, independent non-parametric groups (with Bonferroni correction applied when necessary), and the Independent Groups t-Test was utilized for post-hoc analysis. Univariate and multivariate linear and logistic regression analyses were conducted on significant data confirmed by Spearman correlation. Data were analyzed with a 95% confidence interval, and statistical significance was defined as *p* < 0.05. Analyses were performed using the IBM Statistical Package for Social Sciences-21 (Chicago, IL, USA).

## Results

During the 3-year study period, 123 HAIs occurred in 99 (14.1%) of the 700 ICU hospitalizations (Fig. 1). In 18 cases, multiple HAIs developed, while a single HAI occurred in 81 (11.6%) hospitalizations.

Among the 18 patients with multiple HAIs, the infection combinations were distributed as follows: CAUTI + VAP (*n* = 7), CAUTI + CLABSI (*n* = 2), CLABSI + VAP (*n* = 4), CLABSI + lower respiratory infection (*n* = 1), CLABSI + pneumonia + VAP (*n* = 1), recurrent CLABSI + VAP (*n* = 2), and CAUTI + VAP + CLABSI + pneumonia (*n* = 1). In total, 10 CAUTIs occurred within the multiple infection subgroup.

DA-HAIs were observed in 63 (9%) of these 81 cases. Specifically, 26 (3.7%) were VAPs, 25 (3.6%) were CLABSIs, and 12 (1.7%) were catheter-associated CAUTIs. Klebsiella pneumoniae was the most frequently isolated microorganism across all infection types. Among the 700 hospitalizations, 384 (54.6%) involved male patients, and 292 (41.7%) were elderly. Community-acquired infections were present in 241 (34.4%) cases, while 284 (40.6%) hospitalizations involved HAIs prior to ICU admission. The mortality rate was 32.7%, with 229 patients dying (Table 1). Additionally, 85% of hospital admissions were made through emergency or other service departments.

**Table 1:** General characteristics according to HAI status by PS matching

			Before PS Matching			After PS Matching											
			HAI		p (OR)	HAI		p (OR)	VAP		p (OR)	CLABSI		p (OR)	CAUTI		p (OR)
			(+)	(-)		(+)	(-)		(+)	(-)		(+)	(-)		(+)	(-)	
			N = 99	N = 601		n = 81	n = 81		n = 26	n = 26		n = 25	n = 25		n = 12	n = 12	
Male			58(58.6)	324(53.9)	0.39	50(61.7)	40(49.4)	0.12	20(76.9)	18(69.2)	0.54	14(56)	13(52)	0.78	5(41.7)	8(66.7)	0.32
Age		[years]*	60.5 ± 18	57.8 ± 19	0.21	62 ± 18.4	59.2 ± 17	0.24	68.4 ± 16	65.5 ± 16	0.32	56.2 ± 20	56.2 ± 20	0.79	57.1 ± 15	54.3 ± 21	0.93
		≥65	45(45.5)	247(41.1)	0.44	40(49.4)	33(40.7)	0.27	18(69.2)	17(65.4)	0.77	11(44)	10(40)	0.78	4(33.3)	6(50)	0.51
LoS		ICU (days)*	38.6 ± 28	10.2 ± 11	**<0.001**	33.9 ± 20	17.7 ± 17	**<0.001**	44.3 ± 22	21.2 ± 22	**<0.001**	28 ± 16.4	20.8 ± 21	**0.02**	27.8 ± 16	14.9 ± 11	**0.05**
		Hospital(days)*	42.7 ± 28	20.9 ± 35	**<0.001**	37.8 ± 21	27.4 ± 22	**<0.001**	48.2 ± 21	25.9 ± 23	**<0.001**	31.5 ± 19	30.9 ± 25	0.44	35.1 ± 16	26.2 ± 17	0.24
ICU pre-infection (days)*			16.4 ± 12	10.2 ± 11	**<0.001**	17.2 ± 12	17.7 ± 17	0.26	20.7 ± 13	21.2 ± 22	0.18	15.4 ± 11	20.8 ± 21	0.73	17.3 ± 12	14.9 ± 11	0.63
Hospital pre-infection(days)*			20.7 ± 13	20.9 ± 35	**<0.001**	21.1 ± 13	27.4 ± 22	0.27	24.6 ± 13	25.9 ± 23	0.44	18.9 ± 14	30.9 ± 25	0.08	24.6 ± 12	26.2 ± 17	0.84
MV		**Mechanic** **Ventilator**	99(100)	478(79.5)	**<0.001** *(1.2)*	81(100)	81(100)	1	26(100)	26(100)	1	25(100)	25(100)	1	12(100)	12(100)	1
		[days]*	27.4 ± 18	9.3 ± 10	**<0.001**	26 ± 18	14.7 ± 15	**<0.001**	32.4 ± 19	17.5 ± 19	**0.001**	21.6 ± 18	16.1 ± 19	**0.04**	26.8 ± 16	11.8 ± 9	**0.01**
CVC		**Central Venose** **Cathater**	99(100)	503(83.7)	**<0.001** *(1.2)*	81(100)	81(100)	1	26(100)	24(92.3)	0.15	25(100)	25(100)	1	12(100)	12(100)	1
		[days]*	32.6 ± 20	10.5 ± 10	**<0.001**	30.9 ± 20	16.4 ± 15	**<0.001**	37.6 ± 19	18.8 ± 21	**<0.001**	25.4 ± 17	18.1 ± 19	**0.01**	29.4 ± 14	14.2 ± 10	**0.006**
IUC		Intraurethral Catheter (IUC)	99(100)	579(96.3)	0.06	81(100)	81(100)	1	26(100)	26(100)	1	25(100)	25(100)	1	12(100)	12(100)	1
		[days]*	33.1 ± 19	10 ± 10	**<0.001**	31.4 ± 20	16.2 ± 15	**<0.001**	39.5 ± 22	18.5 ± 20	**<0.001**	26 ± 17	17.6 ± 18	**0.004**	30 ± 15	13.3 ± 10	**0.004**
In hospital deaths			55(55.6)	175(29.1)	**<0.001***(3)*	46(56.8)	38(46.9)	0.21	17(65.4)	15(57.7)	0.57	14(56)	14(56)	1	7(58.3)	5(41.7)	0.51
Previous surgery			33(33.3)	181(30.1)	0.52	27(33.3)	23(28.4)	0.5	14(53.8)	10(38.5)	0.27	4(16)	6(24)	0.48	4(33.3)	2(16.7)	0.51
HAI in pre-ICU period			43(43.4)	241(40.1)	0.58	33(40.7)	38(46.9)	0.43	10(38.5)	12(46.2)	0.58	8(32)	8(32)	1	9(75)	9(75)	1
CAI			41(41.4)	200(33.3)	0.14	33(40.7)	33(40.7)	1	9(34.6)	8(30.8)	0.77	16(64)	11(44)	0.16	2(16.7)	1(8.3)	0.76
Comorbidity			29(29.3)	220(36.6)	0.74	72(88.9)	76(93.8)	0.27	22(84.6)	25(96.2)	0.16	23(92)	24(96)	0.56	11(91.7)	11(91.7)	1
Comorbidities	[count]*		2.6 ± 1.7	2.1 ± 1.4	**0.004**	2.7 ± 1.7	2.4 ± 1.5	0.4	2.7 ± 1.9	2.8 ± 1.5	0.74	2.8 ± 1.8	2.3 ± 1.4	0.38	2.6 ± 1.7	1.8 ± 1.1	0.24
	**0**		12(12.1)	66(11)	**0.007**	9(11.1)	5(6.2)	0.9	4(15.4)	1(3.8)	0.31	2(8)	1(4)	0.64	1(8.3)	1(8.3)	0.3
	**1–2**		37(37.4)	323(53.7)		30(37)	39(48.1)		7(26.9)	10(38.5)		10(40)	14(56)		6(50)	9(75)	
	**≥3**		50(50.5)	212(35.3)		42(51.9)	37(45.7)		15(57.7)	15(57.7)		13(52)	10(40)		5(41.7)	2(16.7)	
	Neoplasia		29(29.3)	220(36.6)	0.16	25(30.9)	26(32.1)	0.87	10(38.5)	13(50)	0.41	8(32)	6(24)	0.53	2(16.7)	7(58.3)	0.09
	**Respiratory disease**		14(14.1)	42(7)	**0.02***(2.2)*	13(16)	12(14.8)	0.83	4(15.4)	4(15.4)	1	3(12)	2(8)	0.64	4(33.3)	0	0.18
	**Cardiovascular disease**		31(31.3)	123(20.5)	**0.02***(1.8)*	27(33.3)	18(22.2)	0.12	9(34.6)	11(42.3)	0.57	8(32)	7(28)	0.76	4(33.3)	1(8.3)	0.32
	**Neurological disease**		26(26.3)	103(17.1)	**0.03***(1.7)*	17(21)	18(22.2)	0.85	5(19.2)	6(23.1)	0.74	6(24)	5(20)	0.74	2(16.7)	4(33.3)	0.51
	Urinary disease		24(24.2)	106(17.6)	0.12	19(23.5)	13(16)	0.24	4(15.4)	6(23.1)	0.49	6(24)	6(24)	1	4(33.3)	1(8.3)	0.32
	**Hypertension**		37(37.4)	162(27)	**0.03***(1.6)*	33(40.7)	26(32.1)	0.26	13(50)	7(26.9)	0.09	13(52)	7(28)	0.09	1(8.3)	4(33.3)	0.32
	**Diabetes Mellitus**		34(34.3)	150(25)	**0.05***(1.6)*	30(37)	27(33.3)	0.62	9(34.6)	11(42.3)	0.57	10(40)	6(24)	0.23	4(33.3)	1(8.3)	0.32
	Solid organ transplant		3(3)	23(3.8)	0.7	3(3.7)	3(3.7)	1	1(3.8)	0	0.32	0	2(8)	0.15	1(8.3)	0	0.76
	Bone-marrow transplant		4 (4)	21(3.5)	0.79	4(4.9)	2(2.5)	0.41	2(7.7)	0	0.15	1(4)	2(8)	0.56	0	0	1
Risk Factor			99(100)	552(91.8)	**0.03***(0.85)*	81(100)	80(98.8)	0.32	26(100)	26(100)	1	25(100)	24(96)	0.32	12(100)	12(100)	1
Ri s k Fa c t o r s	[count]*		5.4 ± 2.4	3.1 ± 2	**<0.001**	5.2 ± 2.2	4.7 ± 2.1	0.23	5.8 ± 2.5	5.1 ± 2.6	0.34	4.8 ± 2	5 ± 2.2	0.63	5 ± 1.9	4.3 ± 1.8	0.38
	**0**		0	49 (8.2)	**<0.001**	0	1(1.2)	0.3	0	0	0.51	0	1(4)	0.77	0	0	0.62
	**1–3**		20(20.2)	313(52.1)		18(22.2)	22(27.2)		5(19.2)	7(26.9)		6(24)	5(20)		2(16.7)	3(25)	
	**≥4**		79(79.8)	239(39.8)		63(77.8)	58(71.6)		21(80.8)	19(73.1)		19(76)	19(76)		10(83.3)	9(75)	
	**Nasogastric Tube**		83(83.8)	255(42.4)	**<0.001***(7)*	69(85.2)	70(86.4)	0.82	23(88.5)	22(84.6)	0.69	20(80)	22(88)	0.45	10(83.3)	10(83.3)	1
	**Blood Transfusion**		43(43.4)	156(26)	**<0.001** *(2.2)*	31(38.3)	28(34.6)	0.63	12(46.2)	9(34.6)	0.4	7(28)	8(32)	0.76	6(50)	6(50)	1
	**Dialysis**		23(23.2)	88(14.6)	**0.03***(1.8)*	19(23.5)	21(25.9)	0.72	5(19.2)	3(11.5)	0.45	5(20)	8(32)	0.34	4(33.3)	2(16.7)	0.51
	**Immuno-suppression^**		42(42.4)	136(22.6)	**<0.001** *(2.5)*	34(42)	35(43.2)	0.87	12(46.2)	13(50)	0.78	15(60)	14(56)	0.78	3(25)	1(8.3)	0.51
	**Decubitus ulcer^**		26(26.3)	58(9.7)	**<0.001** *(3.3)*	21(25.9)	17(21)	0.46	10(38.5)	10(38.5)	1	5(20)	5(20)	1	1(8.3)	0	0.76
	**Chest Tube**		12(12.1)	36(6)	**0.03***(2.2)*	10(12.3)	11(13.6)	0.82	4(15.4)	5(19.2)	0.72	1(4)	2(8)	0.56	2(16.7)	0	0.51
	**Peripheral Artery Catheter**		87(87.9)	374(62.2)	**<0.001** *(4.4)*	71(87.7)	56(69.1)	**<0.001** *(3.2)*	23(88.5)	18(69.2)	0.09	21(84)	19(76)	0.48	11(91.7)	10(83.3)	0.76
	**Enteral Nutrition**		54(54.5)	157(26.1)	**<0.001** *(3.4)*	46(56.8)	48(59.3)	0.75	13(50)	16(61.5)	0.41	15(60)	16(64)	0.77	8(66.7)	7(58.3)	0.76
	Parenteral Nutrition		2(2)	17(2.8)	0.65	2(2.5)	5(6.2)	0.25	1(3.8)	0	0.32	0	2(8)	0.15	0	2(16.7)	0.51
	**Tracheostomy**		47(47.5)	58(9.7)	**<0.001** *(8.5)*	35(43.2)	12(14.8)	**<0.001** *(4.4)*	16(61.5)	7(26.9)	**0.01** *(4.3)*	9(36)	4(16)	0.11	4(33.3)	2(16.7)	0.62
	**CPR**		8(8.1)	19(3.2)	**0.02***(2.7)*	5(6.2)	4(4.9)	0.73	1(3.8)	2(7.7)	0.56	2(8)	0	0.15	1(8.3)	0	0.76
	**PEG**		16(16.2)	19(3.2)	**<0.001** *(5.9)*	7(8.6)	6(7.4)	0.77	3(11.5)	2(7.7)	0.64	1(4)	0	0.32	1(8.3)	0	0.76
Invasive RF Count			457(84.9)	1,648(90)	**<0.001** *(0.62)*	362(86)	318(83)	0.25	127(85)	107(82)	0.42	95(80)	101(82)	0.75	56(93.3)	51(98.1)	0.23
Antibiotic Use			99(100)	582(96.8)	0.07	81(100)	79(97.5)	0.5	26(100)	26(100)	1	25(100)	25(100)	1	12(100)	12(100)	1
Patient Isolation in ICU			92(92.6)	183(30.4)	**<0.001** *(30)*	74(91.4)	36(44.4)	**<0.001** *(13.2)*	25(96.2)	14(53.8)	**<0.001** (21.4)	23(92)	9(36)	**<0.001** *(20.4)*	10(83.3)	4(33.3)	**0.04** *(10)*

CAI: Community-aquired Infection // CPR: Cardiopulmonary Resuscitation // PEG: Percutaneous Endoscopic Gastrostomy *:*[Mean±SD] //***^:** non-Invasive

**Fig. 3:**
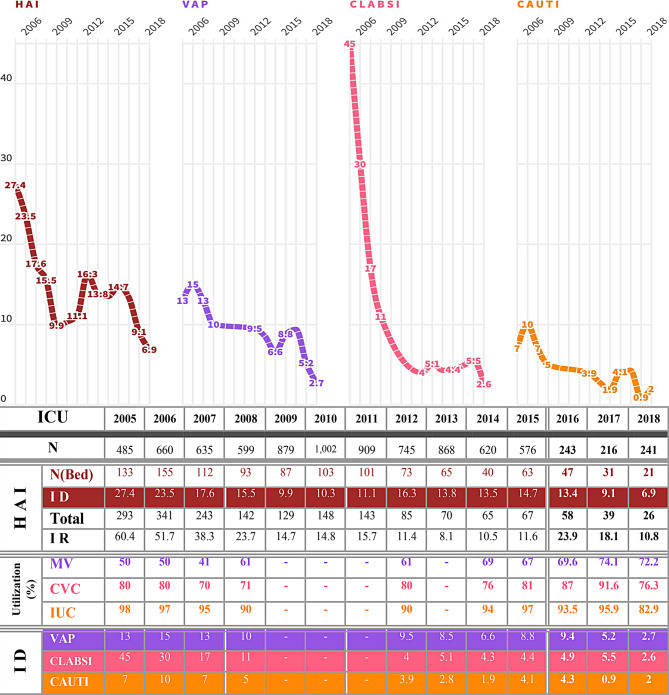
Incidence density (ID) and incidence rate (IR) distribution of HAI surveillance of the ICU

Although there has been a steady decline in the incidence densities of VAP and CLABSI in the ICU, fluctuations in the incidence density of CAUTI and device utilization rates have been observed. Over the past three years, the HAI incidence rate (IR) decreased from 23.9% to 10.8%, and the incidence density (ID) dropped from 14.7‰ to 6.9‰ (Fig. 3).

**Fig. 4:**
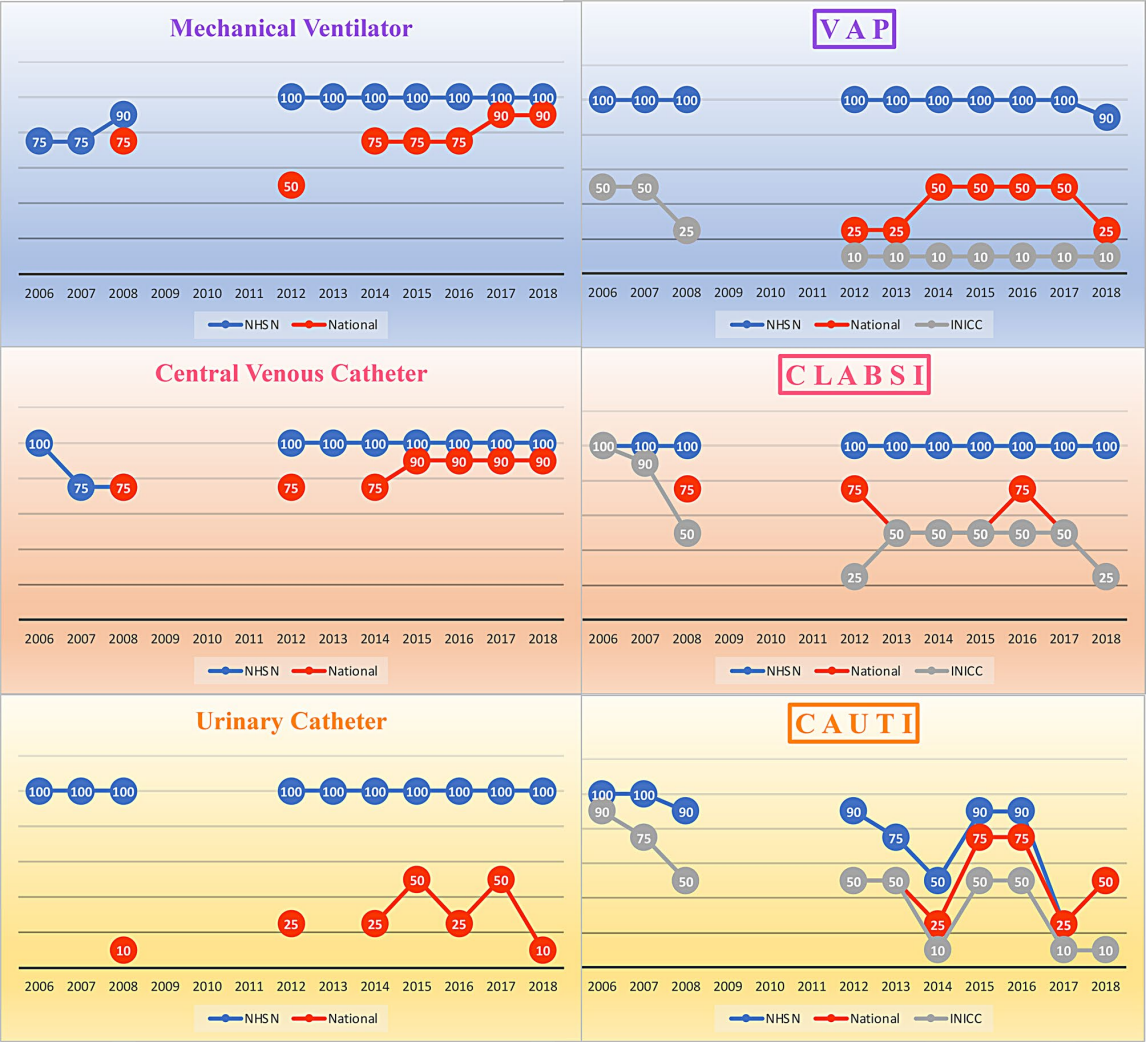
Percentiles of DA-HAIs are developing dependent on device utilization rates in the ICU, according to national, NHSN and INICC standards

While the incidence densities of VAP and CLABSI are within the 90^th^-100^th^ percentiles of NHSN standards (indicating the highest levels), they correspond to lower percentiles in both national and INICC standards (Fig. 4).

Antibiotics were administered to all patients with HAIs, while only 19 patients (3.2%) without HAIs did not receive antibiotics.

The total direct cost for an ICU patient with an HAI was found to be $10,540. Among DA-HAIs, VAP, CAUTI, and CLABSI ranked in descending order in terms of cost and additional financial burden. VAP led to the largest cost increase, raising expenses by 147% and adding $6,781 in costs. While most cost increases were statistically significant, the cost increase associated with CLABSI was not statistically significant.

## Discussion

**Fig. 5:**
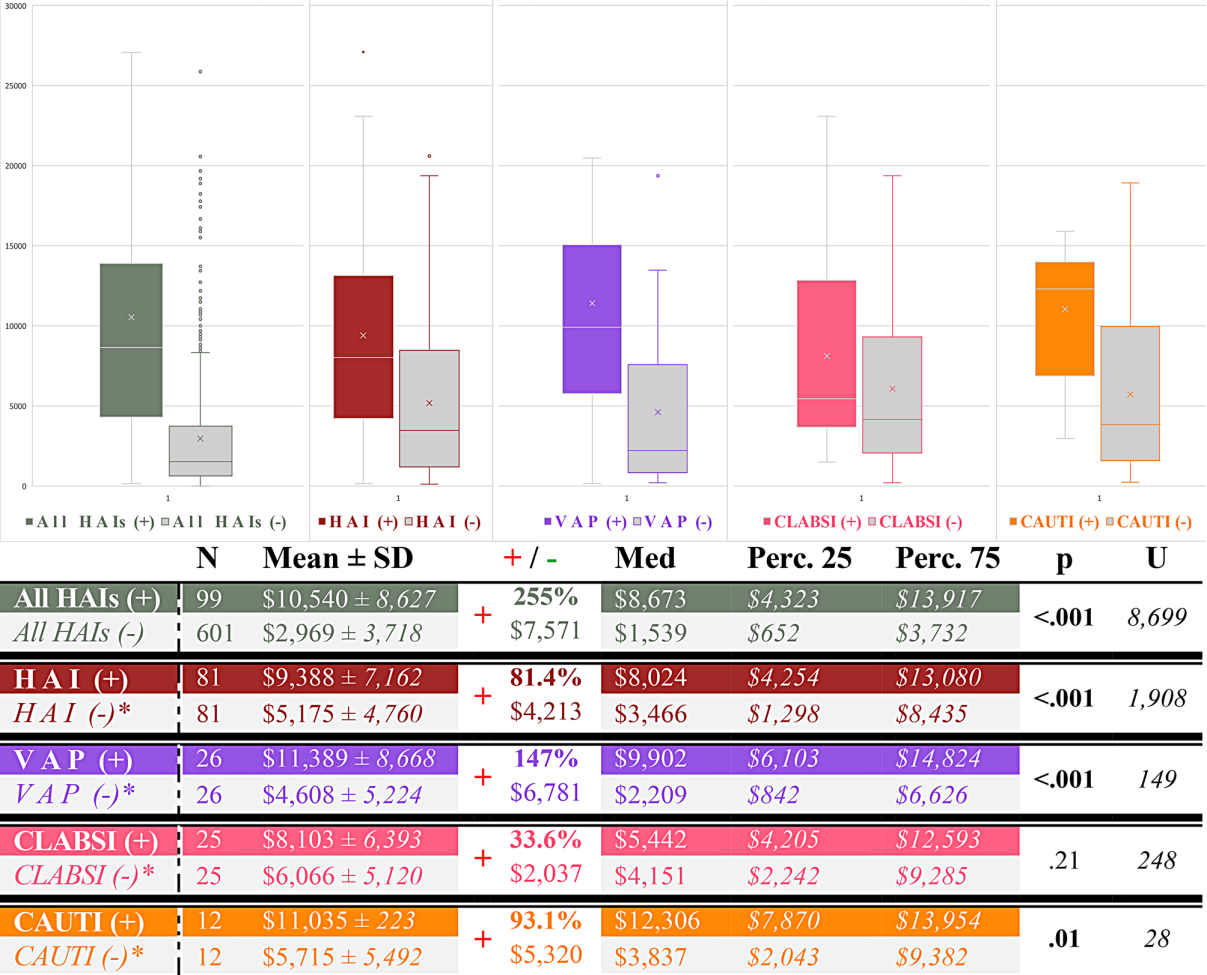
Direct hospitalization costs ($) based on DA-HAIs and HAIs. *: PS matching

This study highlights the additional total direct costs generated by DA-HAIs and the factors influencing these costs. We found that the development of a HAI in the ICU resulted in an overall cost increase of 81.4% ($4,213). Specifically, the costs increased by 147% ($6,781) for VAPs, 33.6% ($2,037) for CLABSIs, and 93.1% ($5,320) for CAUTIs. Consequently, the total direct costs were calculated as $9,388 for HAIs, $11,389 for VAPs, $8,103 for CLABSIs, and $11,035 for CAUTIs (Fig. 5).

Previous studies have reported wide variability in DA-HAI–related monetary costs across countries and healthcare systems, reflecting differences in reimbursement models, resource utilization, and methodological approaches. Reported additional costs range from several thousand dollars to more than $40,000 per episode in different settings, with VAP and CLABSI generally associated with higher costs than CAUTI in most reports [21–25].

Studies from Türkiye similarly demonstrate heterogeneous cost estimates, largely depending on study design and costing methodology [26–30]. Given these substantial inter-country differences, direct comparison of absolute monetary values is challenging. Therefore, our interpretation focuses primarily on infection-specific excess costs and associated length of stay rather than direct currency-based comparisons.

The upward trend in costs, particularly when comparing Türkiye to high-income countries, can be explained by the relatively lower healthcare service costs in developing countries [31]. Given the variability in daily ICU costs across healthcare systems, excess ICU length of stay may provide a more comparable measure of economic burden than absolute monetary values alone. Although CAUTI is generally associated with lower attributable costs in the literature, the relatively higher cost estimates observed in our cohort may reflect subgroup-specific factors such as small sample size, higher comorbidity burden, and prolonged ICU stay. Therefore, these findings should be interpreted cautiously. Additionally, as hospital-related errors are minimized in developed countries, HAIs occur more frequently in patients with multiple risk factors, leading to higher average costs per patient. The inclusion of multiple infections and the absence of matching techniques like PSM in previous studies may also explain these disparities.

The primary cost drivers contributing to additional direct costs in our study were ICU LoS and isolation measures (*p* < 0.05). Specifically, the increase in LoS due to all HAIs was 91.5% (16.2 days), while the increases for VAP, CLABSI, and CAUTI were 109% (23.1 days), 34.6% (7.2 days), and 86.6% (13.9 days), respectively (Table 1). A comprehensive meta-analysis by Durand-Zaleski et al. assessed 40 ICU studies, reporting additional LoS due to HAI to range from 5 to 21 days [21]. Our findings of 7 to 23 additional days are consistent with these results. In a study from Argentina by Rosenthal et al., additional LoS due to DA-HAI was reported as 83% for VAP, 117% for CLABSI, and 45.8% for CAUTI [32]. In comparison, our study found these values to be 109%, 34.6%, and 86.6%, respectively. In Zimlichman et al.‘s meta-analysis, additional LoS was determined to be 8.4 days for VAP and 6.9 days for CLABSI [24], closely aligning with our CLABSI results. The increased LOS observed in VAP patients likely reflects greater underlying illness severity and clinical vulnerability, rather than unit-level ventilator utilization alone (Fig. 4). In contrast, Duszynska et al. reported LoS increases of 21 days for VAP, 24 days for CLABSI, and 23 days for CAUTI [25], further supporting that extended LoS contributes to increased average costs. Residual imbalance in comorbidity burden within the CAUTI subgroup may partly explain the relatively higher cost estimates and should therefore be considered when interpreting subgroup-specific findings

The HAI incidence density in this study was 6.9‰, with specific densities of 5.9‰ for VAP, 4.5‰ for CLABSI, and 2.4‰ for CAUTI (Fig. 3). In the Polish study by Duszynska et al., these rates were 6.5‰, 12.6‰, and 1.8‰, respectively [25]. In contrast, Zimlichman et al.‘s meta-analysis reported 1.3‰ for VAP, 1.9‰ for CLABSI, and 1.3‰ for CAUTI [24]. While CAUTI rates in our study are relatively aligned, our institution showed higher incidence densities (IDs) for VAP and CLABSI compared to averages from developed countries. The initially high CLABSI rate followed by a subsequent decline may reflect progressive maturation of infection prevention practices, increasing adherence to central line care bundles, and improved familiarity with surveillance definitions over time rather than a single identifiable intervention. The relatively lower frequency of CAUTI may reflect a greater clinical focus on more severe device-associated infections such as VAP, potentially influencing detection and reporting patterns.

Notably, the NHSN percentiles for our institution fell within the 75th–90th percentile for VAP, 90^th^–100^th^ for CLABSI, and 25^th^–50^th^ for CAUTI (Fig. 4). These findings suggest that while our CAUTI rates are lower than other DA-HAIs, there is significant room for improvement in reducing overall HAI rates. Therefore, the prevention of DA-HAIs in our ICU should remain a top priority. Although percentile data in the literature is sparse [33, 34], our study is a pioneering effort for Türkiye, providing valuable insights and setting a precedent for future research.

Although the present study reflects the 2016–2018 period, we believe the findings remain clinically and epidemiologically relevant because detailed propensity score–matched analyses evaluating the economic burden of DA-HAIs from middle-income healthcare systems remain limited in the literature. Furthermore, because the study period predates the COVID-19 pandemic, the cohort may provide a valuable baseline reference for future comparisons regarding ICU infection epidemiology, healthcare resource utilization, and infection-control strategies.

The PSM technique, a post-randomization analytical approach, was used to match common variables between independent patient groups, thereby reducing confounding and selection bias. PSM has been widely utilized in previous studies evaluating the economic impact of healthcare-associated infections [7, 35–37]. Although its application in device-associated HAI cost analyses remains limited, similar methodological approaches have been reported in VAP research; for example, Xiaomeng Yi et al. applied PSM to improve comparability between ventilated patient groups [6].

To our knowledge, only a limited number of studies have used PSM specifically to evaluate DA-HAI–attributable costs in ICU settings. The most comparable study to ours was conducted by Duszynska et al., which investigated DA-HAI–related costs but did not employ PSM [25]. Therefore, our study adds to the literature by applying a propensity score framework to better control confounding when estimating the economic burden of DA-HAIs.

Our study also revealed a notably high level of antibiotic use in the control group, indicating potential overuse. This finding raises concerns about antibiotic stewardship and its implications for both HAI outcomes and healthcare costs.

This study has several limitations. First, because of limited temporal generalizability, as it reflects data from a specific time period and may not capture temporal variations in infection patterns or healthcare practices. Therefore, the findings should be interpreted within this context. In particular, changes in infection-control practices, antimicrobial stewardship policies, ICU management strategies, healthcare reimbursement systems, and post-pandemic healthcare dynamics since 2018 may affect the present-day applicability and interpretation of the reported incidence and cost estimates. Additionally, although our sample size was sufficient for the primary analyses, larger multicenter studies across different time periods would strengthen the generalizability of these findings. Second, only direct medical costs were analyzed. Indirect costs, fixed hospital costs, and alternative economic evaluations such as opportunity costs were not included, potentially leading to an underestimation of the overall economic burden. A formal cost-effectiveness framework incorporating QALY or DALY metrics was not applied, which may limit interpretation from a health economics perspective. Furthermore, although all costs were originally calculated in Turkish Lira (TRY) according to the official reimbursement tariffs of the Social Security Institution during the study period (2016–2018) and subsequently converted to US dollars (USD) for international comparability, the reported values represent nominal costs. Adjustments for inflation, purchasing power parity, or currency fluctuation were not performed. In addition, as the cost data reflect the 2016–2018 period, substantial increases in healthcare expenditures over the past decade may limit direct temporal comparisons with studies conducted in earlier or more recent years. International cost comparisons should therefore be interpreted with caution. The study could also have benefited from a more granular breakdown of cost components and the systematic inclusion of validated comorbidity indices such as the Charlson Comorbidity Score. In addition, standardized severity-of-illness measures such as ASA, APACHE, or SAPS were not systematically available for the entire cohort and therefore could not be incorporated into the regression or propensity score models. The absence of these validated severity indices may represent a source of residual confounding. Lastly, incomplete or missing surveillance data—particularly the absence of ICD codes for primary diagnoses and predefined archival gaps during institutional transitions—may have influenced the robustness of risk adjustment. In addition, because updated NHSN percentile distributions were not available beyond 2013, the use of fixed 2013 benchmark thresholds may not fully capture potential temporal shifts in international reference distributions. Following completion of the study, feedback was provided to the hospital’s Infection Control Committee (HICC) to improve future data completeness and surveillance quality.

The strength of this study lies in its novel approach, as it is one of the few cost analyses to examine both HAI and DA-HAI in conjunction within an ICU setting and the first to employ PSM to analyze these costs.

In conclusion, we found that DA-HAIs prolong intensive care stays and impose substantial costs. According to NHSN standards, our institution’s ICU ranked among those with the highest incidence of DA-HAIs. Although important steps have been taken in the last 20 years regarding the control and prevention of HAIs in Türkiye, and specifically in our institution, it is evident that further progress is required. It is essential to mitigate excessive antibiotic use and maintain more consistent follow-up notes to enhance patient outcomes. To our knowledge, this is among the first studies to apply propensity score matching for estimating the direct costs of device-associated healthcare-associated infections in an ICU setting, highlighting the substantial economic burden associated with these infections and potential areas for improvement in infection control.

## Electronic supplementary material

Below is the link to the electronic supplementary material.

Supplementary Material 1

## Data Availability

The datasets generated and analyzed during the current study are available from the corresponding author upon reasonable request.

## Code availability

The codes used throughout the study are available from the corresponding author upon reasonable request.
