# Supplementary material for: Device-associated healthcare-associated infections in an intensive care unit in Türkiye: trends and additional costs - a retrospective propensity matched cohort analysis
Source: BMC Health Serv Res. 2026 Jun 3;26:1090. doi: 10.1186/s12913-026-14833-3 (PMC13459249; doi:10.1186/s12913-026-14833-3)
Supplement: Supplementary file 1 [file 12913_2026_14833_MOESM1_ESM.pdf]

Is isolation applied?

T.C.
ISTANBUL UNIVERSITY
ISTANBUL FACULTY OF MEDICINE
Hospital Infection Control Committee

HOSPITAL INFECTION SURVEILLANCE FORM FOR INTENSIVE CARE UNITS

THIS SECTION WILL BE FILLED FOR ALL PATIENTS

1. Service name/bearing number :...

2. Patient's name, surname / T.C No :...

3.Age (to be specified in months under 2 years old) :.....................

4.Gender (K / E) :..................... **5.Birth** weight if newborn:............

6.Date of hospitalization :......../......../201.. **7.** Date of admission to the unit :......../......../201..

8. Date of completion of the form :......../......../201.. **9.Date** of discharge :......../........./201..

10. Diagnosis (if any, the diagnosis code will also be written)
1-...
2-...
3-...

11. Community-acquired infection at admission to the Intensive Care Unit
(0) None — If any, please specify ....
(1) Yes — Microorganism name...

12. Hospital-acquired infection on admission to the Intensive Care Unit
(0) None — If any, please specify ....
(1) Yes — Microorganism name...

13.From which hospital/service did the patient come? ....

14.Use of antibiotics in the intensive care unit

	Antibiotic name - dosage	Start date	Expires	Notes
a) Use of antibiotics for therapeutic purposes	……………………………………<br>……………………………………<br>……………………………………<br>……………………………………<br>……………………………………<br>……………………………………<br>……………………………………<br>……………………………………<br>……………………………………	…………………<br>…………………<br>…………………<br>…………………<br>…………………<br>…………………<br>…………………<br>…………………<br>…………………	…………………<br>…………………<br>…………………<br>…………………<br>…………………<br>…………………<br>…………………<br>…………………<br>…………………	……………………………<br>……………………………<br>……………………………<br>……………………………<br>……………………………<br>……………………………<br>……………………………<br>……………………………<br>……………………………<br>……………………………
b) Use of antibiotics for prophylactic purposes	……………………………………<br>……………………………………<br>……………………………………<br>……………………………………	…………………<br>…………………<br>…………………<br>………………...	…………………<br>…………………<br>…………………<br>…………………	……………………………<br>……………………………<br>……………………………

ITF HİKK

15. Invasive procedures performed while lying in the intensive care unit

**Process**	**History**	**Days**
Mechanical Ventilation		
Urinary tract catheter		
Central venous catheter		
Umbilical catheter		

16. Underlying diseases

(1) Acute abdomen
(2) Asthma Bronchiole
(3) Benign solid tumor
(4) Renal impairment
(5) Skin diseases
(6) Diabetes
(7) Other endocrine disorder
(8) Other GI diseases
(9) Other Heart Diseases
(10) Other chronic lung diseases. (other than TM)
(11) Other neurological disorder
(12) GI bleeding
(13)Pregnancy complications
(14)Hemotous malignant
(15)Hernias
(16)HIV/AIDS
(17)Hypertension
(18) Immune mechanism disease.
(19) Inflammatory bowel disease.
(20)Heart failure
(21)Bone marrow transplant
(21)COPD
(23)Congenital abnormalities
(24)Coronary artery disease
(25)Chronic liver disease
(26)Malabsorbtion
(27)Malignant solid tümör
(28)Non-malignant hematogenous disease.
(29)Diseases of the pancreas
(30)Primary immunodeficiency
(31)Psychiatric disorders
(32)Rheumatic valve disease.
(33)Diseases of the gallbladder
(34)Cerebral palsi
(35)Cerebrovascular accident
(36)Solid organ transplantation
(37)Urogenital system disease.
(38)Presence of foreign body / prosthesis

17.Risk factors

(1) Surgical drain
(2) Angio
(3) Maternal diabetes
(4) Maternal infection
(5) AV-Fistül/ şant
(6) Unconsciousness
(15)ERCP
(16)Premature rupture of membranes
(17)External safra drenajı
(18)General body trauma
(19)Chest tube
(29)Krause
(30)Karamni'i
(31)Lumbar Puncture
(32)Lumbar/ventricular drainage catheter
(33)Meconium aspiration
(43)Peritoneal Dialysis
(44)Rektoskopi / Rektosigmoidoskopi
(45)Systofix
(46)Sistoscopy
(47)Respiratory failure
(48)Torosentez

(7) Bronchoscopy	(20)H2 receptor antagonist	(34)Nazal entübasyon	(49) Total Parenteral Birth (TPN)
(8) Multiple Pregnancy	(21)Hemodialysis	(35)Nasogastric tube	(50)TPN (with SVK)
(9) Decibustus	(22)İmmunsupressure	(36)Nefrostomi	(51)Trakeostomes
(10) Low apgar score	(23)Internal bile drainage	(37)Nötroper	(52)Transfusion
(11) Low birth weight	(24)Intraaortic balloon pump	(38)Parasper	(53)Ürodinami
(12) Articulate	(25)Indwelling central catheter	(39)PEG	(54)Ventricular shunt
(13) Endoscopy	(26)Cardiac catheterization	(40)Peripheral species catheter	(55)Foreign body
(14)Interal Beslenme	(27)Cardio Pulmonary Respiratory.	(41) Peripheral venous catheter	(56)Burnt
	(28)Colonoscopes	(42)Pericardial tube	

THIS SECTION WILL BE FILLED IN IF THE PATIENT HAS UNDERGONE AN OPERATION

18. Date of operation: ..../......../201...
19. Name of the operation (the largest if more than one) :..
20. Classification of wounds : (1) Clean (2) Clean - contaminated (3) Contaminated (4) Dirty
21. Type of operation: (1) Elective (2) Emergency

**22. Patient's ASA score (physical condition before the operation):**	
1) Normal, healthy patient	4) Patient with a systemic disease that causes life-threatening failure
2) Patient with mild systemic disease	5) Terminally ill patient who is not expected to live for 24 hours whether the operation is performed or not
3) Patient with severe systemic disease, but not life-threatening insufficiency	

THIS SECTION WILL BE FILLED IF THE PATIENT HAS AN INFECTION

23. Hospital infection in the patient : (0) None

If there is a nosocomial infection , the localization code is:	**Date of determination**
(1) Urinary tract infection	......./ ..... / 201..
(2) Surgical site infection	......./ ..... / 201..
(3) Pnömon (□PNÖ1 □PNÖ2 □PNÖ3 □VIP)	......./ ..... / 201..
(4) Bloodstream infection	......./ ..... / 201..
(5) Bone and joint infection	......./ ..... / 201..
(6) Central nervous system infection	......./ ..... / 201..
(7) Cardiovascular system infection	......./ ..... / 201..
(8) Eye, ear, nose, throat or mouth infections	......./ ..... / 201..
(9) Gastrointestinal tract infection	......./ ..... / 201..
(10) Lower respiratory tract infection (except pneumonia)	......./ ..... / 201..
(11) Reproductive system infections	......./ ..... / 201..
(12) Skin and soft tissue infection	......./ ..... / 201..
(13) Systemic infections	......./ ..... / 201..

NOTE: If there are any microorganisms that reproduce and whose names are written below, only their resistance status to the relevant antibiotic will be stated in question 24.

(1) Acinetobacter sp.	Carbapenem (Imipenem, meropen)
(2) Entecigarettes	Vancomycin
(3) Koagülaz negatif stafilokok	Methicillin
(4) E.coli	3rd generation cephalosporin (ceftriaxone, cefotaxime, ceftazidime)
(5) K.pneumoniae	3rd generation cephalosporin (ceftriaxone, cefotaxime, ceftazidime)
(6) K.pneumoniae	Carbapenem (Imipenem, meropen)
(7) P.aeruginosa	Carbapenem (Imipenem, meropen)
(8) P.aeruginosa	Quinolone (Cyproxacin or ofloxacin)
(9) P.aeruginosa	3rd generation cephalosporin (ceftazidime, cefaperazone/sulbactam) and cefepime
(10) Enterobacter sp.	3rd generation cephalosporin (ceftriaxone, cefotaxime, ceftazidime) and cefepime

**24. a- Infectious microorganisms:**	**24. b- Infectious microorganisms:**
Infection localization code:	Infection site code: ....
Material protocol no..	Material protocol no.................................
Cinsi material..	Cinsi material..
1-...	1-..
2-...	2-..

25.Fate of the Patient

(1) Hospital infection was treated and discharged
(2) Discharge while the infection is ongoing
(3) Transport to another ward while the infection continues- (service..............................)
(4) Transferred to another ward after treatment – (ward..............................)
(5) Death due to nosocomial infection
(6) Death from other causes

ITFHİKK

Communication
Istanbul University Faculty of Medicine, Hospital Infection Control Committee Basic Medical Sciences 3. Kat Çapa / İSTANBUL
Tel: 0212 414 20 00 / 31116 – 33150 0212 414 21 12 e.mail:itfhikk@istanbul.edu.tr
